# Repeat-Dose Toxicity Study Using the AFPL1-Conjugate Nicotine Vaccine in Male Sprague Dawley Rats

**DOI:** 10.3390/pharmaceutics11120626

**Published:** 2019-11-23

**Authors:** Reynaldo Oliva, Nya L. Fraleigh, Jordan D. Lewicky, Mildrey Fariñas, Tamara Hernández, Alexandrine L. Martel, Ingrid Navarro, García-Rivera Dagmar, Reinaldo Acevedo, Hoang-Thanh Le

**Affiliations:** 1 Finlay Institute of Vaccine, Havana 11600, Cuba; reyolivacuba@gmail.com (R.O.); mfarinas@finlay.edu.cu (M.F.); thernandez@finlay.edu.cu (T.H.); inavarro@finlay.edu.cu (I.N.); dagmar.garcia@gmail.com (G.-R.D.); racevedo7810@gmail.com (R.A.); 2 Health Sciences North Research Institute (HSNRI), Sudbury, ON P3E 2H3, Canada; nfraleigh@hsnri.ca (N.L.F.); jlewicky@hsnri.ca (J.D.L.); amartel@hsnri.ca (A.L.M.); 3 Northern Ontario School of Medicine (NOSM), Laurentian University, Sudbury, ON P3E 2C6, Canada; 4 Chemistry & Biochemistry and Biology Departments, Laurentian University, Sudbury, ON P3E 2C6, Canada

**Keywords:** nicotine vaccine, AFPL1 adjuvant, repeat-dose toxicity, heterologous administration

## Abstract

Tobacco smoking is the cause of 20% of Canadian deaths per year. Nicotine vaccines present a promising alternative to traditional smoking cessation products, but to date, no vaccine has been able to move through all phases of clinical trials. We have previously demonstrated that the AFPL1-conjugate nicotine vaccine does not induce systemic or immunotoxicity in a mouse model and that a heterologous vaccination approach is more advantageous than the homologous routes to inducing mucosal and systemic anti-nicotine antibodies. The purpose of this study was to confirm the safety profile of the vaccine in a repeat-dose toxicity study. The heterologous vaccination strategy was again used, and Sprague Dawley rats were administered a dose five times greater than in our previous studies. Physiological conditions, food and water consumption, body temperature, injection site inflammation, relative weights of organs, histopathology, and blood chemistry and hematology were evaluated during the course of the vaccination period to determine the safety of the vaccine. The AFPL1-conjugate nicotine vaccine did not induce clinically relevant changes or induce symptoms that would be associated with toxicity, making it a promising candidate for future investigations.

## 1. Introduction

Tobacco smoking continues to be both a social and economic burden in Canada, resulting in approximately one out of every five deaths and 16.2 billion dollars in both direct and indirect costs per year [1]. Moreover, the growing use of E-cigarettes, particularly among young adults, will surely add to the number of individuals who become addicted to nicotine and represents a potentially serious problem given the unknown long-term health effects of these devices [2]. The continued low rate of success from the currently available nicotine cessation products, including pharmacotherapeutics [3], in breaking nicotine addiction has fueled research to develop alternative therapies for addiction. For years, a nicotine vaccine has been pursued as an alternative. Using a strong adjuvant to allow the immune system to recognize the otherwise nonimmunogenic nicotine would produce antibodies that are able to bind to it and prevent it from crossing the blood–brain barrier to reinforce the nicotine addiction.

We have been developing a conjugate-nicotine vaccine that combines the hapten with the adjuvant Finlay proteoliposome (AFPL1) nanoparticle adjuvant [4]. Using a heterologous vaccination strategy, where the initial priming event consisted of an intramuscular (IM) and an intranasal (IN) vaccination with two subsequent IN boosts, the vaccine formulation was able to significantly induce anti-nicotine antibodies in both the lung and in the sera of mice, resulting in two levels of protection [5]. Additionally, the antibodies were able to bind to nicotine in the lung and in the blood when the mice were challenged in vivo with nicotine, as demonstrated by competitive ELISAs for IgA and IgG [5]. Both the homologous and heterologous routes did not show signs of toxicity when assessing daily physiological conditions and the gross necropsy of the organs [5].

Toxicological studies are a critical aspect of the vaccine development process. Repeat-dose toxicity evaluations in a single animal species with increased doses are the standard means by which the non-clinical safety profile of a vaccine is established [6,7,8]. The rat is a versatile and well characterized model in vaccine toxicology studies [6]. In particular, the adult Sprague Dawley (SD) rat is considered a relevant biological model and has been used extensively to evaluate both intrinsic toxicity and toxicity associated with the immune responses induced by vaccines [9,10,11]. Parameters monitored during repeat-dose studies typically include food and water consumption, body weight, and temperature [6,7,8]. Endpoint parameters are more extensive and include assessing blood chemistry, hematology, organ weights with macroscopic assessments, as well as histopathology on a range of tissues including pivotal organs such as the lung, brain, liver, and spleen [6,7,8].

In the present study, we continued the incremental development of our AFPL1-conjugate nicotine vaccine and investigated its toxicity in a repeat-dose study using an SD rat model. Importantly, we continued to utilize the heterologous vaccination strategy to assess the safety of the vaccine with this alternative administration route. While doses are usually only doubled when moving from mice to rats [12], we increased the dose of the vaccine to be 5 times greater than what was administered to BALB/c mice in previous studies [4,5]. In addition, we also increased the number of boost events from two to three. The rats were evaluated for physiological criteria such as food and water consumption, weight gain, body temperature before and after each vaccination event, swelling at the injection site, hematology and blood chemistry variables, macroscopic evaluation of the organs and their relative weights, and histopathology of primary organs 3, 7, and 14 days after the final dose. To the best of our knowledge, this is the first preliminary report evaluating the toxicity of a nicotine vaccine candidate using multiple doses and combined routes of administration.

## 2. Materials and Methods

### 2.1. Animals and Husbandry

Male SD rats were purchased from the National Center for the Production of Laboratory Animals (Havana, Cuba) and were housed in Tecniplast^®^ rat cages at the Animal Care Facility at the Finlay Institute of Vaccine Research. Rats were provided specialized feed for rodents, and the water used was provided in acidified water bottles (750 mL). Both food and water were available ad libitum. The animal room was maintained at a temperature of 21 ± 2 °C and a relative humidity of 55 ± 5%. These parameters were recorded daily in addition to maintaining a 12 h light and dark cycle. Rats were randomly placed into groups of 15, with 5 animals per cage, and allowed to acclimatize to their surroundings for one week prior to the commencement of the experimental protocol. All protocols were approved by the ethics committee of the Finlay Institute of Vaccine Research (28/08/2017; Code: 2-2017-7).

### 2.2. Vaccine and Vaccination Protocol

The vaccine was prepared as previously described [5]. Rats were immunized using a heterologous vaccination strategy (Table 1). The initial vaccination involved a simultaneous administration via both the IM and IN routes. Three weeks after the first dose, 3 subsequent IN vaccinations followed as per the schedule in Table 1. The duration of the repeat-dose study was 10 weeks (71 days). Three vaccination groups were assigned for the study, including animals immunized with phosphate-buffered saline (PBS) as a negative control, or AFPL1 alone to ensure that there were no negative effects associated with the adjuvant. A 50 μg dose of the nicotine conjugate vaccine was used for all vaccinations (based on nicotine concentration) but was administered in different volumes suitable to the route of administration; the intramuscular dose volume of 200 µL was administered as 100 µL in each leg, while the intranasal dose volume of 80 μL was delivered as 40 μL per nare.

### 2.3. Clinical Observations and Symptoms

All observations started from the experimental time zero (*T*_0_), which was considered as the day of the first vaccination event. Animals were monitored daily, paying special attention to the administration site and the development of any physical conditions such as difficulty moving (limp), piloerection, prostration, involuntary movements, shaking of the head, ataxia, salivation, nasal secretions/irritation, sneezing, difficulty breathing, tearing, hyperactivity or lethargy, incoordination, or diarrhea. Weight (g) and food (g/animal/day) and water consumption (mL/animal/day) were measured weekly as measures of toxicity.

The body temperature of the rats was measured using a laser clinical thermometer (Equate^®^, non-contact forehead thermometer, model #10857, Walmart, Mississauga, ON, Canada) directed towards the right ear. Body temperature was measured before and after each inoculation at 24 h intervals for 72 h.

Muscle diameter was evaluated as previously described for rats [13] with a digital caliper (Electronic Caliper with digital display, 6′′, 150 mm, Mastercraft, Toronto, ON, Canada) by measuring the diameter of the inoculated limb at the center of the musculature of the thigh region. Leaving the teeth of the caliper on the inner and external side of the muscle, the caliper was closed without putting pressure on the leg. This was done both before and after the IM vaccination at 24 h intervals for 72 h.

### 2.4. Euthanasia and Blood Collection

Rats were euthanized on days 3, 7, and 14 after the last vaccination by an overdose of the general anesthetic sodium thiopental (80 mg/kg of animal weight, Quimefa^®^, Havana, Cuba, Lot: 092017) and cardiac puncture. Blood was collected and mixed with EDTA as an anticoagulant. All animals were subjected to 4 h of fasting prior to euthanasia and blood collection. Blood was also collected on days 7, 21, 42, and 57 for anti-nicotine IgG ELISAs.

### 2.5. Hematological and Blood Biochemical Evaluation

Total red and white blood cell counts were analyzed using an automatic hematological analyzer (Mindray BC-2800Vet, Shenzhen, China). Parameters reported included hemoglobin (HBG), hematocrit (Hto), leukocyte totals (LT), polymorphonuclear cells (PMN), lymphocytes (Lf), eosinophils (E), and monocytes (M). Blood biochemistry was analyzed using diagnostic kits (CENTIS Diagnostics, Havana, Cuba). Parameters measured included glucose, urea nitrogen, uric acid, creatinine, alkaline phosphatase (ALP), total protein (TP), triglycerides, cholesterol, direct bilirubin, creatine phosphokinase (CPK), alanine aminotransferase (ALT), and aspartate aminotransferase (AST). One normal and one pathological control serum sample (CENTIS Diagnostics) were analyzed for every group of 10 test samples as a quality control measure.

### 2.6. Anatomopathological Studies and Organ Weights

The anatomopathological studies (gross necropsy) were performed immediately after the euthanasia of each animal. All organs and sites of vaccine administration were examined macroscopically.

Solid or parenchymal organs (brain, heart, lung, spleen, liver, and kidneys) and thymus were removed and weights recorded. They are expressed as relative weight (RW) and were calculated by the following equation:

RW = (OW × 100)/EEW,(1)

where OW is the organ weight and EEW is the animal end weight on the day of euthanasia.

Tissue samples were processed by fixation in 4% formaldehyde neutralized with calcium carbonate until embedding in paraggin. Sections (4–6 μm thickness) were taken using a microtome (Histolide 2000, Leica Biosystems, Wetzlar, Germany). Tissue slices were stained with a hematoxilin–eosin (HE) mix (QUIMEFA, Cuba) and observed using conventional microscopes (CH-2, Olympus, Tokyo, Japan). The results were compared between all of the vaccinated groups.

### 2.7. Immunotoxicological Evaluation

Systemic inflammation was assessed by measuring the total area of the spleens of the animals by groups using ImagJ software Version 1.43. Organ histology for the brain, cerebellum, lung, liver, and spleen was also assessed.

### 2.8. Anti-Nicotine ELISAs

Anti-nicotine IgG antibodies in sera samples were measured by an indirect ELISA using MaxiSorp (Nunc) microtiter plates as previously described [4]. Briefly, biotinylated goat anti-rat IgG was used as secondary antibody to detect the nicotine-specific systemic antibodies in the sera obtained over the course of the vaccination protocol. The plates were incubated with pNitrophenyl phosphate (pNPP, >97%, Sigma Aldrich, St. Louis, MO, USA), and the reaction was stopped with 3N NaOH after 30 min. The plates were read at an optical density (OD) of 405 nm with a subtraction of 490 nm. An internal standard curve for the quantification of anti-nicotine IgG was carried out by pooling the sera from rats (*n* = 10) with the highest anti-nicotine dilution titer. Relative concentrations of IgG were calculated from a 4-parametric standard curve (ELISA for Windows CDC, 2005 software, USA). The standard was assigned a concentration of 100 arbitrary units (AU)/mL for each serogroup, respectively.

### 2.9. Statistical Analysis

Statistical analyses were performed using GraphPad Prism 5. Multiwise group analyses were performed using an ANOVA with a Tukey HSD. Data were considered significant when *p* < 0.05.

## 3. Results

We vaccinated rats with our previously published conjugate AFPL1 nicotine vaccine to assess toxicity throughout the vaccination protocol. No clinical symptoms were observed during the vaccination protocol using a more concentrated dose (5 times more concentrated than previous studies), nor were there any deaths of vaccinated animals from any of the groups mentioned.

**Figure 1:**
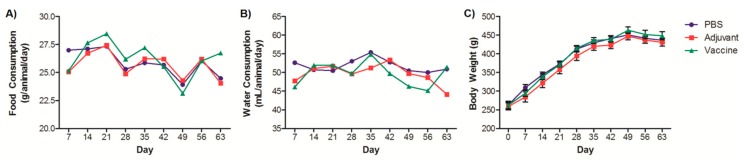
Clinical observations of rats vaccinated with the nicotine vaccine candidate via the heterologous route. (**A**) & (**B**) Food and water consumption were monitored throughout the vaccination protocol. Each value represents consumption relative to all animals in the groups. (**C**) Rats were weighed throughout the vaccination protocol. Each value represents the average ± SEM of all animals in each group.

Food and water consumption were evaluated on weekly intervals over the span of the vaccination protocol (Figure 1A,B). The average weekly consumption of water was 50.71 ± 9.03 mL and of food was 26.33 ± 4.04 g. There were no significant differences between the groups of rats for either consumption of food or water. This consumption is similar to that reported for the species and reported by us in other studies [13,14,15,16,17,18]. Accordingly, the body weight of the rats increased over the course of the study (Figure 1C). The weight-increase curves between the vaccinated groups and controls did not show any statistical differences and are on par with other published growth curves [15,19,20,21]. Body weight has been commonly considered as a sensitive indicator of toxicity of xenobiotics in a wide range of toxicological studies [20,21,22,23]. Toxicity usually manifests as either a decrease in weight gain compared to controls or absolute losses of body weight.

**Figure 2:**
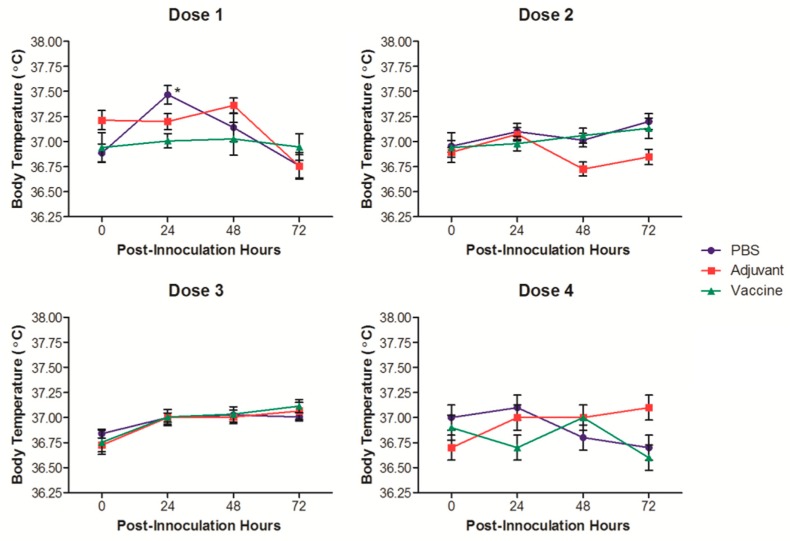
Temperature measurements of rats vaccinated with the nicotine vaccine candidate via the heterologous route. Corporal temperatures of the rats were measured before and each day after vaccinations for 3 days. Each value represents the average ± SEM of all animals in each group. * *p* ≤ 0.05 as compared to the pre-vaccination measurement.

The normal physiological body temperature range reported for SD rats is 35.9 to 37.5 °C [24]. The temperature of the rats in this study was followed for 3 days after each vaccination (Figure 2). The temperatures of the rats were all within the normal physiological range, and there were no increases that could be considered a fever. The only statistically significant temperature increase, although not clinically relevant, occurred in the PBS control group 24 h after the first vaccination event.

**Figure 3:**
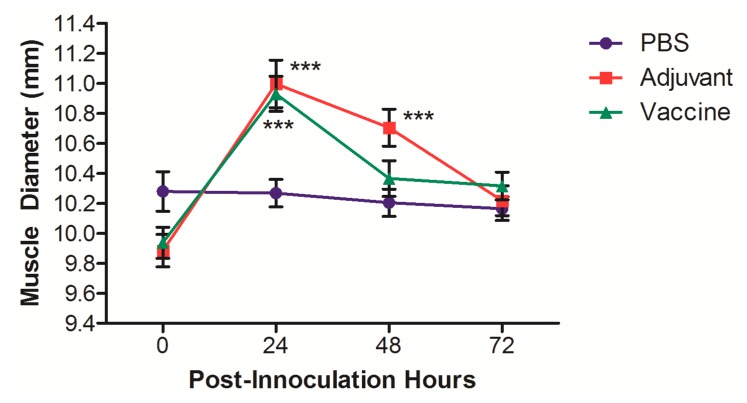
Muscle diameter measurements of rats vaccinated with the nicotine vaccine candidate via the heterologous route. The muscle diameters of both hind legs were measure for each rat before and each day after the intramuscular (IM) vaccination for 3 days. Each value represents the average ± SEM of both legs for all animals in each group. *** *p* ≤ 0.001 as compared to the pre-vaccination measurement.

In order to assess the inflammation induced by the vaccine when administered IM, the muscle diameter of the legs of the rats was measured after receiving the IM vaccination as part of the initial heterologous vaccination event (Figure 3). A significant increase in muscle diameter 24 h after vaccination was noted in the rats receiving either the adjuvant control or the vaccine; however, these increases were only transient, and muscle diameters returned to normal by 72 h after the vaccination. The changes in muscle diameter did not result in changes in locomotion or obvious signs of pain/distress.

**Figure 4:**
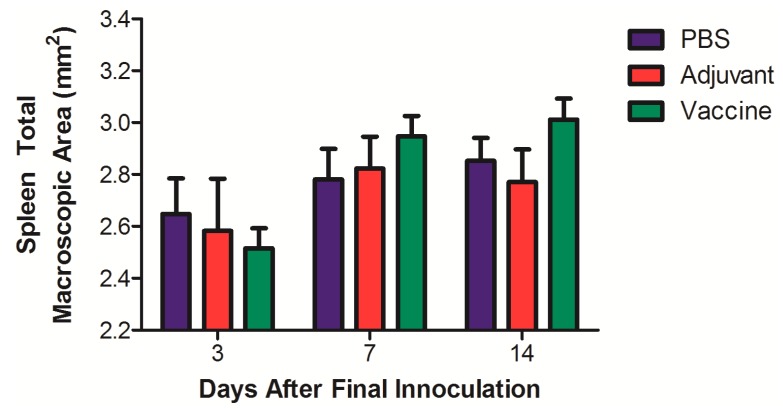
Morphometric evaluation of total macroscopic area of spleens of rats vaccinated with the nicotine vaccine candidate via the heterologous route. Rats were sacrificed at different time-points after their final vaccination and spleen areas analyzed. Values represent the average ± SEM of the 5 animals in each group per time-point.

**Figure 5:**
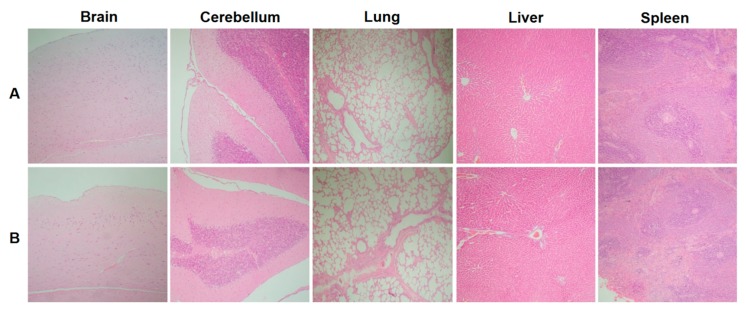
Histological study of organ morphology of rats vaccinated with the nicotine vaccine candidate via heterologous route. Various organs were harvested from rats vaccinated with either (**A**) Phosphate-buffered saline (PBS) or (**B**) the nicotine vaccine candidate for histological analysis (HE stain) 7 days after the final vaccination. A 100× magnification was used.

Macroscopic studies performed on all organs and systems for each of the rats studied did not show any lesions that would suggest acute or chronic toxicity. There were no perceptible local changes at the sites of administration. When the relative organ weights for each animal were evaluated, there were no significant differences observed between any of the groups for any organ (Table 2). A macroscopic morphometric evaluation of spleen diameters showed no significant differences between the different groups in the total areas of this organ (Figure 4). Key organs were subjected to a histological evaluation, and no differences in inflammation, changes in morphology, or recruitment of leukocytes were noted between the control and the vaccine group (Figure 5).

The blood collected from the rats at the time of euthanasia was subjected to hematological (Table 3) and blood chemistry analyses (Table 4a,b) in order to evaluate parameters that could be associated with toxicity. No significant differences were observed between the control groups and the vaccinated group for any of the parameters measured. In the blood, the primary leukocytes measured were neutrophils and lymphocytes, which is similar to other toxicological studies evaluating adjuvants using rats [25].

**Figure 6:**
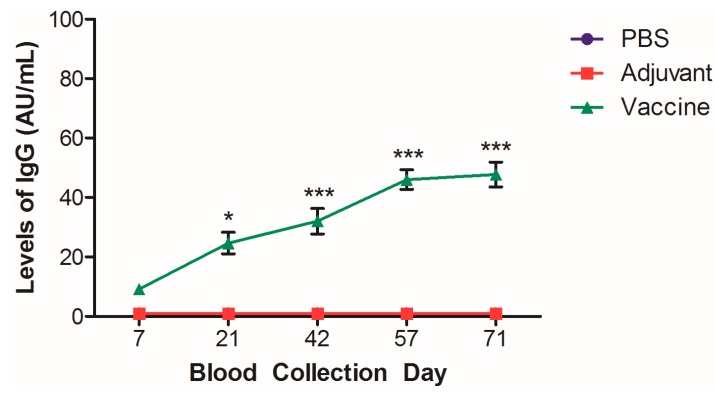
Levels of systemic anti-nicotine IgG in the sera of rats. Sera was collected from the blood of rats vaccinated with the nicotine vaccine candidate and analyzed by ELISA. *N* = 5–7, ± SEM *** *p* < 0.001 and * *p* < 0.05 as compared to day 7 measurement.

The sera that was collected from blood was analyzed for levels of systemic anti-nicotine IgG. Levels of anti-nicotine IgG were detectable as early as 7 days after the first vaccination event (Figure 6), and significant levels of anti-nicotine IgG were detected at each collection point thereafter, plateauing by day 57 as compared to day 7. Similar to our previously published results, the control groups did not show cross-reactivity and non-specific results from the established ELISA protocol.

## 4. Discussion

Smoking continues to be a worldwide epidemic, with over 8 million deaths attributed to tobacco use per year [26]. The World Health Organization estimates that up to half of all lifetime smokers of tobacco will die as a result of smoking; therefore, developing a novel and effective anti-nicotine vaccine is crucial. We previously published the AFPL1-conjugate nicotine vaccine, demonstrating that it is able to bind to nicotine and prevent it from entering the brain in a mouse model [4]. To the best of our knowledge, we were the first group to report a nicotine vaccine that induced both mucosal (IgA, IgG) and systemic anti-nicotine antibodies [5] with no preliminary signs of toxicity. The next step in the development of this vaccine was to evaluate its safety in a SD rat model using the heterologous vaccination strategy and a higher dose, which was done at the Finlay Institute of Vaccine in Cuba.

Toxicity can be assessed by investigating physiological and pathological parameters to determine local and systemic issues that arise from repeated vaccination events. IN vaccines, in particular, need to be rigorously tested because of the ability of IN-delivered vaccines to induce transient Bell’s Palsey (facial nerve paralysis), which continues to be a major challenge for the development of IN-delivered vaccines such as that for influenza [27]. AFPL1 has been used extensively as part of adjuvant and vaccine development at the Finlay Institute of Vaccine in Cuba and is multi-adjuvanted with the ability to activate Toll-like receptors (TLRs) 2, 4, and 9 (reviewed by [28]). AFPL1 is derived from the safe and clinically used VA-MENGOC-BC^®^ for protection from *Neisseria meningitis* B and has been used IN [29,30,31] as part of the institute’s mucosal vaccine development. The cochleate, AFCo1, derived from AFPL1, has been shown to be devoid of toxicity when administered repeatedly in SD rats [32]. Because this vaccine is preventative and therapeutic for nicotine addition, adverse side effects are not acceptable [27] and safety evaluations need to be performed, despite the adjuvant having already been established as safe in the literature when delivered either IN or IM.

Overall, the heterologously delivered IN/IM AFPL1-conjugate nicotine vaccine demonstrated no overt signs of toxicity when assessing physiological conditions. Signs of IN toxicity would have resulted in behavioral differences with changes in food and water consumption [33], which were not seen over the duration of the trial. Similar to our previous investigations, we did not see clinically relevant increases in temperature. The only group that showed any changes was the negative control group, and in order for there to be an established fever, the body temperature would need to be above 38 °C. Not surprisingly, we saw transient but significant increases in muscle diameter 24 and 48 h after vaccinating with either the adjuvant or the vaccine. This transient increase may be due to the recruitment of leukocytes at the site of injection, similar to other injectable adjuvants [34], and would not be considered a safety issue.

Immunotoxicity was evaluated by hematology and morphometric analysis of the spleens 3, 7, and 14 days after the final vaccination. The hematological measurements of the blood showed no significant changes, suggesting no acute or chronic systemic immunological changes as compared to the controls. Hematological measurement in rats is a relative measure of immunotoxicity that can be used to determine the health of the animal over time [35]. This is also supported by the morphometric analysis of the spleen. If there was systemic inflammation, we would have expected to see size changes in the spleen associated with inflammation; however, there were no significant changes as compared to the control or the adjuvant alone at any time-point.

Systemic toxicity associated with organ damage was assessed using a blood chemistry panel, relative weights, and the macropathology of the major organs 3, 7, and 14 days after the final vaccination. Parameters measured in the blood chemistry analysis are clinically relevant and would show signs of toxicity related to the heart, liver, gall bladder, kidneys, bones, and damage to either the muscles or joints. Together, these factors were able to show that, both acutely and chronically, there were no changes that occurred in the organs resulting in changes in relative weight, surface lesions, or changes in baseline levels of commonly measured parameters of a blood chemistry panel. This was also in line with the histopathology of vital organs, including the brain, cerebellum, and liver, which showed no marked differences between the controls and the vaccinated groups. Local toxicity was demonstrated by the histological examination of the lungs, a crucial organ for evaluating the safety of the vaccine since it was delivered IN. The histology of the lungs showed no signs of inflammation, abnormal lung infiltrate, or additional damage as compared to the non-vaccinated controls. Although a limitation of this study is the lack of histology related to the initial site of administration, the nasal passages, the rats demonstrated no overt physiological conditions related to irritation or inflammation of the nares. Due to the volume of administration, the vaccine would have deposited primarily in the lungs, making them the most important local organ of interest. Further in-depth investigations involving the AFPL1-nicotine vaccine will be needed in the future to assess the nasal passages, lymph nodes, lung washes, and other histopathology related to the lungs.

Relative changes in food and water consumption, weight, and hematology and blood chemistry panels are frequently used for determining overall toxicity associated with repeated exposure to various compounds [36,37,38], indicating that the parameters that we have evaluated are in line with other groups. Previous nicotine vaccines, such as NicQb and NicVAX, have been evaluated for their safety during preclinical and clinical trials [38,39,40,41]. Toxicity for these vaccines focused on systemic toxicity and assessing locally where the vaccine was administered, in addition to reactogenicity, when administered to human participants.

Previous investigations of the mouse model for the AFPL1-conjugate nicotine vaccine have demonstrated that the vaccine induces anti-nicotine antibodies [4,5]. However, these models use inbred mice, which represents a limitation for assessing heterogeneic responses that would be expected during preclinical trials. Establishing that we can induce significant levels of anti-nicotine antibodies in outbred SD rats is promising and provides evidence that the vaccine could induce a therapeutic threshold of anti-nicotine antibodies necessary for smoking cessation.

In summary, our pre-clinical evaluation of the AFPL1-conjugate nicotine vaccine is consistent with previous evaluations of the safety of AFPL1 when used as an adjuvant, where the vaccine and the adjuvants are safe when delivered both IN and IM. Our methods of evaluation are also consistent with other investigations that use rodents as models for repeat-dose toxicity. Based on the lack of toxicity and clinically significant changes when comparing the AFPL1-conjugate nicotine vaccine to the negative controls, we can conclude that the dose used is not toxic for male SD rats and continues to be a promising candidate for the development of a successful anti-nicotine vaccine. Further in-depth studies, as outlined above, will need to be performed as part of the preclinical development of this potentially safe and efficacious anti-smoking cessation therapeutic. Additionally, future investigations involving non-human primates would allow for the evaluation of the vaccine with respect to safety and effective dosing in humans.

## Figures and Tables

**Table 1:** Vaccine experimental design and schedule. PBS—phosphate-buffered saline.

Group	Animals, *n*	*n* Euthanized at *x* Days After 4th Dose		
		*x* = 3	*x* = 7	*x* = 14
PBS	15	5	5	5
Adjuvant (AFPL1)	15	5	5	5
Vaccine	15	5	5	5
**Schedule**	**Route**	**Volume**		
Day 0	IM/IN	200 µL (100 µL per leg)/80 µL (40 µL per nare)		
Day 21	IN	80 µL (40 µL per nare)		
Day 42	IN	80 µL (40 µL per nare)		
Day 56	IN	80 µL (40 µL per nare)		

**Table 2:** Relative organ weights (%) of rats vaccinated via the heterologous route. Values represent the mean ± SEM of the 5 animals in each group per time-point. No statistical differences were found between the groups for any organ.

Group	Brain	Thymus	Heart	Left Lung	Right Lung	Liver	Spleen	Left Kidney	Right Kidney
	3 Days After 4th Dose								
PBS	0.47 ± 0.01	0.10 ±0.01	0.32 ± 0.01	0.13 ± 0.01	0.26 ± 0.01	3.01 ± 0.26	0.15 ± 0.01	0.36 ± 0.01	0.36 ± 0.01
Adjuvant	0.47 ± 0.01	0.08 ±0.01	0.30 ± 0.01	0.14 ± 0.01	0.28 ± 0.02	2.91 ± 0.14	0.16 ± 0.01	0.35 ± 0.01	0.35 ± 0.01
Vaccine	0.48 ± 0.01	0.11 ±0.01	0.30 ± 0.01	0.12 ± 0.01	0.24 ± 0.01	2.79 ± 0.14	0.16 ± 0.01	0.35 ± 0.01	0.37 ± 0.02
	**7 Days After 4th Dose**								
PBS	0.47 ± 0.01	0.09 ± 0.01	0.35 ± 0.01	0.13 ± 0.01	0.24 ± 0.01	2.96 ± 0.09	0.16 ± 0.01	0.34 ± 0.01	0.34 ± 0.01
Adjuvant	0.49 ± 0.01	0.10 ± 0.01	0.31 ± 0.01	0.13 ± 0.01	0.24 ± 0.01	2.96 ± 0.20	0.17 ± 0.01	0.39 ± 0.02	0.37 ± 0.02
Vaccine	0.49 ± 0.02	0.12 ± 0.03	0.33 ± 0.03	0.14 ± 0.02	0.26 ± 0.03	2.79 ± 0.20	0.17 ± 0.01	0.34 ± 0.01	0.36 ± 0.02
	**14 Days After 4th Dose**								
PBS	0.49 ± 0.01	0.10 ± 0.01	0.31 ± 0.01	0.13 ± 0.01	0.24 ± 0.01	3.16 ± 0.11	0.17 ± 0.01	0.35 ± 0.01	0.36 ± 0.02
Adjuvant	0.47 ± 0.02	0.10 ± 0.01	0.28 ± 0.01	0.13 ± 0.01	0.24 ± 0.01	2.80 ± 0.14	0.16 ± 0.01	0.36 ± 0.01	0.35 ± 0.01
Vaccine	0.44 ± 0.01	0.09 ± 0.01	0.30 ± 0.01	0.12 ± 0.01	0.22 ± 0.01	3.04 ± 0.10	0.17 ± 0.01	0.35 ± 0.02	0.36 ± 0.02

**Table 3:** Hematological studies of rats vaccinated via the heterologous route. Values represent the mean ± SEM of the 5 animals in each group per time-point. No statistical differences were found between the groups for any parameter. HBG: hemoglobin, Hto: hematocrit, LT: leukocyte totals, PMN: polymorphonuclear cells, Lf: lymphocytes, E: eosinophils, M: monocytes.

Group	HBG (g/L)	Hto (mL/100L)	LT (10^3^ mm)	PMN (%)	Lf (%)	E (%)	M (%)	Platelets (× 10^3^ µL)
	3 Days After 4th Dose							
PBS	154.2 ± 4.5	51.0 ± 1.6	5.2 ± 0.2	31.3 ± 1.7	68.7 ± 1.7	0.0 ± 0.0	0.0 ± 0.0	746.4 ± 33.0
Adjuvant	154.1 ± 6.3	53.3 ± 2.2	5.1 ± 0.2	33.6 ± 3.8	66.1 ± 3.8	0.2 ± 0.3	0.0 ± 0.0	744.2 ± 56.4
Vaccine	157.1 ± 3.6	51.5 ± 1.3	5.0 ± 0.1	30.4 ± 2.3	68.4 ± 1.9	0.5 ± 0.6	0.0 ± 0.0	772.3 ± 51.6
	**7 Days After 4th Dose**							
PBS	155.0 ± 1.5	51.8 ± 0.5	5.2 ± 0.1	35.6 ± 3.2	63.4 ± 3.1	0.0 ± 0.0	0.0 ± 0.0	767.2 ± 43.9
Adjuvant	154.2 ± 2.9	51.3 ± 1.0	5.1 ± 0.2	35.4 ± 2.6	63.5 ± 3.3	0.1 ± 0.1	0.1 ± 0.1	797.0 ± 45.3
Vaccine	157.1 ± 3.1	53.1 ± 1.5	5.2 ± 0.2	38.8 ± 3.9	58.8 ± 3.4	0.1 ± 0.2	0.1 ± 0.2	790.4 ± 44.5
	**14 Days After 4th Dose**							
PBS	157.0 ± 4.1	52.4 ± 1.3	6.4 ± 0.5	34.5 ± 5.1	65.3 ± 5.0	0.0 ± 0.0	0.0 ± 0.0	853.1 ± 29.1
Adjuvant	158.1 ± 4.6	53.5 ± 2.3	5.5 ± 0.2	29.3 ± 4.1	70.0 ± 3.8	0.1 ± 0.4	0.0 ± 0.0	764.0 ± 23.8
Vaccine	156.0 ± 3.9	52.8 ± 1.4	6.3 ± 0.5	33.7 ± 3.4	66.3 ± 3.4	0.0 ± 0.0	0.0 ± 0.0	803.2 ± 30.1

**Table 4:** Blood chemistry analysis of rats vaccinated via the heterologous route. Values represent the mean ± SEM of the 5 animals in each group per time-point. No statistical differences were found between the groups for any parameter. (**a**) ALT: alanine aminotransferase, AST: aspartate aminotransferase, TP: total protein. (**b**) CPK: creatine phosphokinase, ALP: alkaline phosphatase.

(**a**)						
**Group**	**ALT** **(UI)**	**AST** **(UI)**	**TP** **(g/dL)**	**Bilirubin** **(mg/dL)**	**Urates** **(µM)**	**Urea** **(mM)**
	**3 Days After 4th Dose**					
PBS	53.67 ± 9.36	167.71 ± 15.38	5.11 ± 0.55	0.07 ± 0.04	64.29 ± 18.31	8.15 ± 0.61
Adjuvant	61.02 ± 12.22	153.65 ± 12.71	5.43 ± 0.42	0.16 ± 0.08	65.94 ± 17.38	8.47 ± 0.91
Vaccine	59.15 ± 10.34	159.43 ± 18.49	6.10 ± 0.85	0.03 ± 0.04	57.86 ± 13.56	8.27 ± 0.89
	**7 Days After 4th Dose**					
PBS	74.38 ± 6.44	163.45 ± 16.50	5.34 ± 0.17	0.01 ± 0.04	69.90 ± 10.55	9.47 ± 1.19
Adjuvant	62.71 ± 9.53	154.88 ± 14.97	5.47 ± 0.40	0.06 ± 0.03	79.13 ± 18.29	8.82 ± 1.23
Vaccine	70.13 ± 11.19	161.26 ± 14.30	5.11 ± 0.38	0.08 ± 0.04	66.31 ± 7.73	8.64 ± 0.93
	**14 Days After 4th Dose**					
PBS	56.45 ± 7.93	128.33 ± 11.69	5.92 ± 0.22	0.01 ± 0.01	87.92 ± 17.11	8.14 ± 1.50
Adjuvant	65.51 ± 13.70	133.53 ± 13.32	5.94 ± 0.41	0.10 ± 0.04	66.27 ± 11.23	8.95 ± 1.25
Vaccine	42.32 ± 6.37	152.44 ± 18.57	6.82 ± 0.77	0.09 ± 0.04	68.32 ± 11.35	9.70 ± 1.03
(**b**)						
**Group**	**CPK** **(UI)**	**Creatinine** **(µM)**	**Glucose** **(mM)**	**ALP** **(UI)**	**Triglycerides** **(mM)**	**Cholesterol** **(mM)**
	**3 Days After 4th Dose**					
PBS	1082.93 ± 201.29	65.12 ± 2.87	7.45 ± 0.52	282.82 ± 17.32	0.39 ± 0.12	1.25 ± 0.18
Adjuvant	1229.24 ± 153.11	68.69 ± 6.18	8.39 ± 0.67	288.32 ± 30.09	0.76 ± 0.24	1.72 ± 0.26
Vaccine	1189.94 ± 80.87	63.16 ± 8.77	9.21 ± 0.80	287.24 ± 16.14	0.94 ± 0.20	1.29 ± 0.10
	**7 Days After 4th Dose**					
PBS	1488.94 ± 173.82	60.83 ± 4.61	9.70 ± 1.70	410.41 ± 38.78	0.85 ± 0.19	1.18 ± 0.19
Adjuvant	1362.28 ± 91.59	61.32 ± 4.97	8.85 ± 1.28	452.10 ± 57.35	0.59 ± 0.14	1.25 ± 0.16
Vaccine	1228.22 ± 49.77	56.60 ± 3.85	10.18 ± 1.22	434.26 ± 33.24	0.63 ± 0.17	0.99 ± 0.13
	**14 Days After 4th Dose**					
PBS	1334.75 ± 150.25	75.83 ± 8.27	10.47 ± 0.89	289.93 ± 20.36	1.17 ± 0.22	1.27 ± 0.11
Adjuvant	1302.22 ± 120.83	78.14 ± 11.44	9.46 ± 1.14	264.22 ± 43.21	1.07 ± 0.30	1.30 ± 0.18
Vaccine	1422.27 ± 134.45	66.49 ± 7.49	10.42 ± 1.12	213.89 ± 22.83	1.51 ± 0.32	1.14 ± 0.09
